# Effect of antidepressants in pregnancy outcomes: A protocol for systematic review and meta-analysis

**DOI:** 10.1097/MD.0000000000027885

**Published:** 2021-12-17

**Authors:** Rixile Hlongwane, Wendy N. Phoswa

**Affiliations:** Department of Life and Consumer Sciences, University of South Africa (UNISA), Science Campus, Private Bag X6, Florida, Roodepoort, South Africa.

**Keywords:** antidepressants, hypertensive disorders of pregnancy, selective serotonin re-uptake inhibitors

## Abstract

**Background:**

Depression is much more common in women pre their pregnancies with antidepressants use less common which is caused by when many patients opt to discontinue with the use due to its side effects it causes. But whether depression is treated by antidepressants or not this has the same negative pregnancy outcomes on both the mother and the unborn and even born child from these mothers.

**Methods:**

Information will be retrieved for this systematic review and meta-analysis study on antidepressants use pregnancy outcomes from PubMed and Google scholar search engines using search medical subjects headings on PubMed and the PICOS framework as the determinant of the research question. All returned articles searched will be stored to Zotero.org and the software RevMan will be used to analyze data.

**Ethics and dissemination:**

The review and meta-analysis will not require ethical approval and the findings will be published in peer-reviewed journals and presented at local and international conferences. In addition, findings from this study will assist in assessing health related risk outcomes of antidepressants during- and postpregnancy on both the fetal and mother either when used pre- and during-pregnancy.

**Systematic review registration:**

International prospective Register of Systematic Reviews (PROSERO) number: CRD42021232111.

## 1 Introduction

Depression is one of the psychiatric disorders already common and prevalent, and it is twice common in women, even before their pregnancies, than in men in the general population and is one of the hardest disease to treat with ∼121 million people globally experiencing antidepressant side effects.^[1]^ With negative side effect experiences because of antidepressant, most patients opt to discontinue treatment which is the cause of relapse in many patients. Antidepressant use prevalence is as less as 20% in pregnant women with 68% who discontinue treatment develop relapse depression.^[2]^ In 2003, antidepressant use prevalence during pregnancies was 13% and 3.1% in White, 1.6% in non-Hispanic, and 0.8% in Hispanic in 2006 to 2010.^[3]^ Depression is ranked number 2 as the leading cause of disability. Women can take a decision to stop taking antidepressant even though in some cases they might stop antidepressants when recommended by their clinicians, whatever the case is, this increases the risk for them to suffer from depression during pregnancy. Whether its occurrence happened pre-pregnancy or during pregnant, in women who are pregnant, it is the leading cause of neonates mortalities and morbidities born from such mothers because of adverse events as low birth weight, pre-term births and small-for-gestation age neonates occurs, when both pre-term births and low birth weights commonly cause their mortalities and morbidities.^[4,5]^ Such adverse events in depressed pregnant women are further increased because of increased cravings and abuse of cigarette smoking and alcohol and inadequate nutritional consumption.

And amongst the factors such as the discontinuation of antidepressant maintenance medication, factors such as stress and hormonal changes are also common in pregnant women who have depression. Hormonal factors which increases neonatal morbidity and mortality adverse events include factors such as the impaired (which is increased) hypothalamic-pituitary-adrenal axis levels and the increment of the sympathetic nervous system hormones such as the corticotropin releasing hormone, cortisol, and catecholamines hormones due to depression. Epigenetic impairments such as DNA methylation is deregulated by antidepressants.^[6]^ And together with deregulated DNA methylation and hypothalamic-pituitary-adrenal axis, placental gene expression, impairments of neurogenesis, are also common. The co-risk factors of depression include a patient's level of education, occupational status, being a single parent with no emotional and practical support as a new mother, as depression during postpartum is also common. Intimate violent partners may be contributing risk factors of depression in pregnant women, being in an unstable mental state as a person/woman may increase the chances of you choosing to be with a partner whose mental health status is also unstable.^[7]^ Intimate violent partners on pregnant women increases the severity of depression and also is associated with postpartum depression incidences.

Antidepressants are also crucial for pregnant women regardless of whether depression is a mild to moderate or severe to prevent both fetus and mother complications, but the problem with antidepressants is that they cause adverse events almost which those that are the same in pregnant women with depression that is not treated.^[6]^ And even though in some studies such as this one pre-term and infant mortality were not found to be associated with antidepressants, but low birth weight as one of the adverse events which are noticed in depressed pregnant women with untreated depression is also noticed in those who are treated with antidepressants.^[6]^ And additionally to these, defects such as the congenital heart defects, new-born adaptation syndrome, and persistent pulmonary hypertension as well as neurodevelopment delay which result in language impairment occurs due to antidepressants. Antidepressants are monoaminergic drugs and produces neurochemical and alters the early development of the brain.

Whether an antidepressants type used during pregnancy will be safer for both the mother and her neonate is still a problem.^[8]^ In some cases, exposed-to-antidepressants-neonates outcomes compared to those not exposed, no significant difference is found, but exposed neonates have autism and behavioral changes symptoms. A study by Prady et al^[8]^ indicates that neonates exposed to selective serotonin re-uptake inhibitors (SSRIs) antidepressants types for example, their neurodevelopment and neurobehavior was statically significant compared to the non-exposed neonates. SSRIs also causes mother's pregnancy-induced hypertension and pre-eclampsia which was seen as the case when many different kinds of SSRIs antidepressants as well as other kinds of antidepressants were used and compared in these patients.^[9]^ Doing this review is therefore important because not only does antidepressants affect a neonate pre-birth but also post its birth, and also the adverse effects experienced by a mother due to antidepressants are serious concern as in some cases postpartum depression as well as the adverse effects as hypertension may still occur also during postpartum.

## 2 Research question

What are the effects of antidepressants in pregnancy outcomes?

## 3 Objectives

a. To assess 3 adverse event, pregnancy-induced hypertension, pre-eclampsia, and eclampsia in pregnant women with depression on antidepressants treatment.
b. To assess the prevalence of adverse outcomes in neonates who are exposed to antidepressants through their mothers.

## 4 Methods

This will be a systematic review and meta-analysis of published studies. This protocol is written in line with the recommendations of the Preferred Reporting Items for Systematic Reviews and Meta-analysis for Protocols (PRISMA-P) guidelines 2015.^[10]^ The results will be reported based on the PRISMA 2015,^[10]^ statement and article screening and selection process will also be demonstrated through a PRISMA-P flow diagram. Furthermore, the current protocol has been registered with the International Prospective Register of Systematic Reviews (PROSPERO): CRD42021232111.

## 5 Eligibility

### 5.1 Study design

This systematic review and meta-analysis will include randomized control trials, cohort, and matched cohort with a clearly defined population and interventions used. While, observational studies, reviews, case studies, and animal studies will be excluded in this study.

### 5.2 Participants

Depressed pregnant women.

### 5.3 Intervention

Antidepressants.

### 5.4 Comparator

Non-depressed pregnant women.

### 5.5 Outcomes

Hypertensive disorders of pregnancy, pre-eclampsia, fetal growth restriction, miscarriage, recurrent spontaneous abortion.

### 5.6 Inclusions criteria

a. Evidence published in the English language.
b. Evidence published between the years 2011 to 2021.
c. Evidence from published global randomized control trial, matched cohort, and cohort studies with antidepressants related pregnancy outcomes/complication, and all of the criteria defining the impact of antidepressants on the incidence of adverse pregnancy and fetal outcomes.

### 5.7 Exclusion criteria

a. Study which does not have the outcomes of interest as objectives.
b. Case reports, expert opinions and review/meta-analysis.
c. Evidence published before the year 2011.
d. Studies evidently published from the anxiety, depressed, or stressed non-pregnant women will also be excluded as antidepressants impact is expected to be evaluated in pregnant women.

### 5.8 Search strategy

The following databases will be searched for eligible studies: Science Direct, Medline, Embase, Pubmed, Africa Wide, Google scholar, ResearchGate, EBSCOhost, Web of Science, and the Cochrane Library, and LILACS. Medical subject headings (MeSH) such as “((((((((((Depressed[All Fields] AND (“pregnant women”[MeSH Terms] OR (“pregnant”[All Fields] AND “women”[All Fields]) OR “pregnant women”[All Fields])) AND (“antidepressive agents”[All Fields] OR “antidepressive agents”[MeSH Terms] OR (“antidepressive”[All Fields] AND “agents”[All Fields]) OR “antidepressive agents”[All Fields] OR “antidepressants”[All Fields])) AND (non-depressed[All Fields] AND (“pregnant women”[MeSH Terms] OR (“pregnant”[All Fields] AND “women”[All Fields]) OR “pregnant women”[All Fields]))) AND (Hypertensive[All Fields] AND (“disease”[MeSH Terms] OR “disease”[All Fields] OR “disorders”[All Fields]) AND (“pregnancy”[MeSH Terms] OR “pregnancy”[All Fields]))) OR (“pre-eclampsia”[MeSH Terms] OR “pre-eclampsia”[All Fields] OR (“pre”[All Fields] AND “eclampsia”[All Fields]) OR “pre-eclampsia”[All Fields])) OR (“fetal growth retardation”[MeSH Terms] OR (“fetal”[All Fields] AND “growth”[All Fields] AND “retardation”[All Fields]) OR “fetal growth retardation”[All Fields] OR (“fetal”[All Fields] AND “growth”[All Fields] AND “restriction”[All Fields]) OR “fetal growth restriction”[All Fields])) OR (“abortion, spontaneous”[MeSH Terms] OR (“abortion”[All Fields] AND “spontaneous”[All Fields]) OR “spontaneous abortion”[All Fields] OR “miscarriage”[All Fields])) OR (Recurrent[All Fields] AND (“abortion, spontaneous”[MeSH Terms] OR (“abortion”[All Fields] AND “spontaneous”[All Fields]) OR “spontaneous abortion”[All Fields] OR (“spontaneous”[All Fields] AND “abortion”[All Fields])))) AND ((“random allocation”[MeSH Terms] OR (“random”[All Fields] AND “allocation”[All Fields]) OR “random allocation”[All Fields] OR “randomized”[All Fields]) AND (“prevention and control”[Subheading] OR (“prevention”[All Fields] AND “control”[All Fields]) OR “prevention and control”[All Fields] OR “control”[All Fields] OR “control groups”[MeSH Terms] OR (“control”[All Fields] AND “groups”[All Fields]) OR “control groups”[All Fields]) AND (“Trials”[Journal] OR “trials”[All Fields]))) OR (“cohort studies”[MeSH Terms] OR (“cohort”[All Fields] AND “studies”[All Fields]) OR “cohort studies”[All Fields] OR “cohort”[All Fields])) OR (Matched[All Fields] AND (“cohort studies”[MeSH Terms] OR (“cohort”[All Fields] AND “studies”[All Fields]) OR “cohort studies”[All Fields] OR “cohort”[All Fields])) and free text searches will be used to search the eligible articles which will be saved to the citation manager Zotero v5.0.81 (Zotero.org). This software will also be used to remove duplicates. The title and abstracts of the articles remaining after exclusion of duplicates will be assessed for eligibility according to the inclusion and exclusion criteria.

### 5.9 Study selection

The full text of all potentially eligible studies will then be reviewed by 2 independent reviewers (RH and WNP), and any disagreement between reviewers with respect to eligible studies for inclusion in the analysis will be assessed for more eligible studies. Initially, studies will be screened by the titles, abstracts, keywords, and synonyms then followed by the identification of the full-text articles. Should discrepancies arise between 2 authors (RH, WNP), a third author will screen such studies, and consensus will be reached through discussion. Zotero v5.0.81 (Zotero.org) will be used to manage extracted data items, including saving relevant and excluded studies with reasons. Importantly, reference lists of included studies will be screened to confirm that no relevant studies are left out. Studies meeting the inclusion criteria will then be subjected to data collection, critical appraisal, risk, and quality evaluation.

## 6 Data management

### 6.1 Data collection process

The reviewers (RH and WNP) will develop a data extraction form that will be used in the collection relevant data items. To reduce data entry errors, selected studies will be independently assessed by 2 reviewers (RH and WNP), the third reviewer will be consulted for arbitration in case of any disagreements.

### 6.2 Data items

Extracted data items will include the author's name, year of publication, gestational age, geographic location, type of antidepressant(s), duration on antidepressant(s), period suffering from depression, other co-morbidity, socioeconomic status: educational background, income status, adverse maternal outcomes: hypertensive disorders of pregnancy, pre-clampsia, adverse fetal outcomes: fetal growth restriction, miscarriage, recurrent spontaneous abortion.

### 6.3 Risk of bias in individual studies

To evaluate the potential risk of bias in randomized control trials, cohort, and matched cohort, Cochrane collaboration tool for assessing bias^[11]^ and Downs and Black checklist^[12]^ will be used. Two independent reviewers (RH and WNP) will appraise all included studies and a third reviewer will be consulted in cases of disagreements.

### 6.4 Data synthesis

A summary of findings table will be used to provide a synthesis of the main outcomes of included studies. Data will be analyzed with Rev Manager (Version 5.3, Cochrane) to conduct a meta-analysis. To measure statistical heterogeneity between studies, I^2^ and chi squared statistical tests will be used.^[13,14]^ An I^2^ value of >50% will be considered substantial heterogeneity.^[15]^ To find the sources of heterogeneity within the included studies, a subgroup analysis and meta-regression comparing the study estimates from different study-level characteristics, quality, intervention type, and the reported effect measure of adverse events will be conducted.

## 7 Quality assessment of the cumulative evidence

The Grading of Recommendations, Assessment, Development, and Evaluation assessment tool^[16]^ will be used to assess the overall quality of evidence. Moreover, the quality of each included study will be independently evaluated by 2 authors (RH, WNP). The third author will adjudicate in cases of disagreements. The quality of evidence will be assessed based on several factors such as study limitations, indirectness of results, and publication or reporting bias. The evidence of each outcome will be rated as high, moderate, low, or very low.

## 8 Discussion

Depression is very common in women of childbearing age and in most cases it occurs prior pregnancy, but the use of certain types of antidepressants during the first term of pregnancy may increase the likelihood of negative pregnancy outcomes especially spontaneous abortion, miscarriages, pre-term birth, hypertension which is likely to progress to pre-eclampsia, and neurodevelopmental impairments during childhood, adolescence, and adulthood in a child born from these mothers, while other antidepressants types do not.^[17]^ Even though discontinuation during the second trimester of pregnancy may limit such outcomes in both mother and fetus, the duration of antidepressant use prior conception is also one of the most important determinant of pregnancy outcomes in mothers with depression.^[17–19]^ Women from disadvantaged socioeconomic backgrounds, those with low educational levels and low household income, are more likely to be affected by depression and more likely to either be exposed to antidepressants until they deliver their babies or to not be exposed at all to antidepressants even if there is a need for them to be.^[17,19]^ It is also known that the type of antidepressant used especially during the recommended period when antidepressants during pregnancy may be used, which is during the first trimester of pregnancy, determines pregnancy outcomes even after discontinuation had happened during the appropriate time during pregnancy.^[20]^ But it is not known how many fetal death occurs because of either being exposed to antidepressant through up to the second trimester of pregnancy or by exposure prior to being pregnant or by being not exposed at all to antidepressants.

The study will address pregnancy outcomes in women who are exposed to antidepressants prior and during pregnancy, especially from during the second trimester of pregnancy whereby most maternal and fetal disorders occur due to these women not discontinuing with antidepressants use during this stage of pregnancy. This systematic review and meta-analysis study will also help investigate the type of antidepressants which are best suitable for use prior pregnancy-conception as well as to assess the prevalence of children's mortalities due to both being exposed to antidepressant and due to a mother having an untreated maternal depression.

## Author contributions

RH and WNP conceptualized, designed, drafted, and approved the final manuscript.

**Conceptualization:** Wendy Phoswa.

**Formal analysis:** Wendy Phoswa.

**Investigation:** Rixile Hlongwane.

**Methodology:** Wendy Phoswa.

**Resources:** Wendy Phoswa.

**Supervision:** Wendy Phoswa.

**Validation:** Rixile Hlongwane.

**Visualization:** Wendy Phoswa, Rixile Hlongwane.

**Writing – original draft:** Wendy Phoswa, Rixile Hlongwane.

**Writing – review & editing:** Wendy Phoswa, Rixile Hlongwane.
